# Impact of heat stress on health and performance of dairy animals: A review

**DOI:** 10.14202/vetworld.2016.260-268

**Published:** 2016-03-12

**Authors:** Ramendra Das, Lalrengpuii Sailo, Nishant Verma, Pranay Bharti, Jnyanashree Saikia, Imtiwati, Rakesh Kumar

**Affiliations:** 1 Department of Animal Genetics & Breeding, Indian Council of Agricultural Research - National Dairy Research Institute, Karnal - 132 001, Haryana, India; 2 Division of Animal Genetics, Indian Council of Agricultural Research - Indian Veterinary Research Institute, Izatnagar - 243 122, Uttar Pradesh, India; 3 Department of Livestock Production & Management, Indian Council of Agricultural Research - National Dairy Research Institute, Karnal - 132 001, Haryana, India; 4 Department of Animal Genetics & Breeding, College of Veterinary Sciences & Animal Husbandry, Agartala - 799 008, Tripura, India; 5 Department of Livestock Production & Management, College of Veterinary Sciences & Animal Husbandry, Agartala - 799 008, Tripura, India

**Keywords:** amelioration, health, heat stress, production, reproduction

## Abstract

Sustainability in livestock production system is largely affected by climate change. An imbalance between metabolic heat production inside the animal body and its dissipation to the surroundings results to heat stress (HS) under high air temperature and humid climates. The foremost reaction of animals under thermal weather is increases in respiration rate, rectal temperature and heart rate. It directly affect feed intake thereby, reduces growth rate, milk yield, reproductive performance, and even death in extreme cases. Dairy breeds are typically more sensitive to HS than meat breeds, and higher producing animals are, furthermore, susceptible since they generates more metabolic heat. HS suppresses the immune and endocrine system thereby enhances susceptibility of an animal to various diseases. Hence, sustainable dairy farming remains a vast challenge in these changing climatic conditions globally.

## Introduction

Stress is a reflex reaction of animals in harsh environments and causes unfavorable consequences ranges from discomfort to death. Climate change is one of the major threats for survival of various species, ecosystems and the sustainability of livestock production systems across the world, especially in tropical and temperate countries. Intergovernmental Panel on Climate Change [1] reported that temperature of the earth has been increased by 0.2°C per decade and also predicted that the global average surface temperature would be increased to 1.4-5.8°C by 2100. It was also indicated that mainly developing countries tend to be more vulnerable to extreme climatic events as they largely depend on climate sensitive sectors like agriculture and forestry [1]. Recently, Silanikove and Koluman [2] also forecasted the severity of heat stress (HS) issue as an increasing problem in near future because of global warming progression.

The thermoneutral zone (TNZ) of dairy animals ranges from 16°C to 25°C, within which they maintained a physiological body temperature of 38.4-39.1°C [3]. However, air temperatures above 20-25°C in temperate climate and 25-37°C in a tropical climate like in India, it enhance heat gain beyond that lost from the body and induces HS [4,5]. As a results body surface temperature, respiration rate (RR), heart rate and rectal temperature (RT) increases which in turn affects feed intake, production and reproductive efficiency of animals. RT >39.0°C and RR >60/min indicated cows were undergoing HS sufficient to affect milk yield and fertility [6]. However, the animal being homeotherms can resist to HS up to some extents depending on species, breed and productivity [7,8]. Among dairy animals, goats were the most adapted species to imposed HS in terms of production, reproduction and also to disease resistance [2]. Studies stipulated that the native breeds survive and perform better as compared to exotic breeds and their crosses under tropical environmental conditions may be due to inability of the exotic genes to express/adapt under tropical conditions [8-10]. Further, the sensitivity of dairy cattle to HS increases with increase in milk production [5], which might be due increase in metabolic heat output with increase production levels in dairy animals. The present review is targeted to collate and synthesize information pertaining HS and its impacts on health, production and reproduction in dairy animals.

## Effects of HS on Health of Dairy Animals

HS effects health of dairy animals by imposing direct or indirect affects in normal physiology, metabolism, hormonal, and immunity system.

### Feed intake and rumen physiology

Increase in environmental temperature has a direct negative effect on appetite center of the hypothalamus to decreases feed intake [11]. Feed intake begins to decline at air temperatures of 25-26°C in lactating cows and reduces more rapidly above 30°C in temperate climatic condition and at 40°C it may decline by as much as 40% [12], 22-35% in dairy goats [13] or 8-10% in buffalo heifers [14]. Reducing feed intake is a way to decrease heat production in warm environments as the heat increment of feeding is an important source of heat production in ruminants [15]. As results, animals experience a stage of negative energy balance (NEB), consequently body weight and body condition score goes down [16].

Increase environmental temperature alters the basic physiological mechanisms of rumen which negatively affects the ruminants with increased risk of metabolic disorders and health problems [17,18]. Nonaka *et al*. [19] reported animal under HS has reduced acetate production whereas propionate and butyrate production increased as rumen function altered. As a response animal consumed less roughages, changes rumen microbial population and pH from 5.82 to 6.03 [20], decreasing rumen motility and rumination [17,18]. Subsequently, affects health by lowering saliva production, variation in digestion patterns and decrease dry matter intake (DMI) [17,18]. Moreover, HS also results into hypofunction of the thyroid gland and effects on metabolism patterns of the animal to reduce metabolic heat production [21].

### Acid-base balance

Animal under HS has increased RR and sweating which results increased body fluid loss that lift up maintenance requirements to control dehydration and blood homeostasis. As RR increases, expiration of CO_2_ via the lungs increases. This results to respiratory alkalosis, as blood carbonic acid concentration decreases [22]. Therefore, animal needs to compensate for higher blood pH by excreting bicarbonate in the urine to maintain the carbonic acid: bicarbonate ratio [23]. Compensation results in urinary bicarbonate loss in an attempt to balance the ratio of carbonic acid to bicarbonate in the blood [21]. Chronic hyperthermia also causes severe or prolonged inappetence which further aggravates the increased supply of total carbonic acid in the rumen and decrease ruminal pH thereby, resulting into subclinical and acute rumen acidosis [15].

### Oxidative stress

Oxidative stress results to increase in reactive oxygen species (ROS) in different cells and tissues of HS animals that have negative impacts on normal physiology and body metabolism. However, body has antioxidants in the form of enzymatic (superoxide dismutase [SOD], glutathione (GSH) peroxidase and catalase), non-enzymatic (albumin, L-cysteine, homocysteine, melatonin and protein sulfhydryl groups), and non-enzymatic low molecular weight antioxidants (ascorbic acid, GSH, uric acid, α-tocopherol, β-carotene, pyruvate and retinol), which increases as a results of HS to provide protection against negative effects of ROS. Significantly (p<0.05) higher levels of stress indices catalase, SOD, GSH reductase, and malondialdehyde was observed in lactating and non-lactating buffaloes [24] and cattle [25] during summer compared to spring seasons. Significantly higher levels of stress indices were believed due to lactation stress since increased milk production predispose to HS-induced oxidative damages in the body [25].

### Immune system

The immune system is the major body defense systems to protect and cope against environmental stressors. Primary indicators of immunity response include white blood cells (WBCs), red blood cells (RBCs), hemoglobin (Hb), packed cell volume (PCV), glucose and protein concentration in blood get altered on thermal stress. WBC (leukocytes) count increase by 21-26% [26] and RBC count decrease by 12-20% [27] in thermally stressed cattle that could be due to thyromolymphatic involution or destruction of erythrocytes. Sejian *et al*. [28] reported highly significant variation of Hb, PCV, plasma glucose, total protein and albumin for the different temperature exposure in malpura ewes. This higher PCV value was an adaptive mechanism to provide water necessary for evaporative cooling process [29]. However, in contrast to these findings reduction of Hb and PCV levels were observed as results of RBC lysis either by increased attack of free radicals on its membrane or inadequate nutrient availability for Hb synthesis as the animal consumes less feed or decreases voluntary intake upon HS [30]. The blood glucose significantly decreased in HS dairy cows in accordance to greater blood insulin activity [9,31]. Release of plasma cortisol increases in stressed animals which causes down-regulation or suppression of L-selectin expression on the neutrophils surface [32]. Further, this poor L-selectin expression responsible for weak neutrophils function by failing to move into the tissue being invaded by pathogens and resulted clinical outcome of disease following exposure to an infective organism [33]. Increase circulating cortisol also causes increase cellular levels heat shock proteins (HSPs) that function as a danger signal to the immune system to encourage increased killing of pathogenic bacteria by neutrophils and macrophages against invading bacteria [34]. Hence, regular assessment of blood constituents is useful in appraisal of the health status of animals in hot-humid regions.

Health problem like subclinical or clinical ketosis [16] and higher risk of liver lipidosis and impairs liver function [35] were encountered. Sanders *et al*. [36] observed that lameness incidence increases with an increase in air temperature that could be due to increase in standing time [37]. Further, lameness causes thin soles, white line disease, ulcers, and sole punctures [36] and increases the likelihood for early culling from the herd. Climate change may bring about substantial shifts in disease distribution and outbreaks of disease prevalence in previously unexposed animal populations possibly with the breakdown of endemic stability [38]. Changes in rainfall and temperature regimes may affect both the distribution and the abundance of disease-causing vectors was also reported by Thornton *et al*. [38]. In accordance, Dhakal *et al*. [39] also observed incidence of external parasite as the first ranked (43.3%) problems in the warm temperate. The increase in THI resulted in increased incidence of mastitis in cows (p<0.01) while Murrah buffaloes were less affected [40]. Further incidence was more in Sahiwal and Tharparkar cows (p<0.01) than the crossbred Karan Swiss and Karan Fries cows [40]. Higher incidence of mastitis in dairy cows could be due to high temperatures facilitating survival and multiplication of pathogens carrier fly population associated with hot-humid conditions. Excess heat load in extreme cases not only compromises animal welfare but also results into death of the animals [2,41].

## Effect of HS on Production and Reproduction Performance of Dairy Animals

### Milk production and composition

HS adversely affects milk production and its composition in dairy animals, especially animals of high genetic merit [42-46]. Berman [7] estimated that effective environmental heat loads above 35°C activate the stress response systems in lactating dairy cows. In response dairy cows reduce feed intake which is directly associated with NEB, which largely responsible for the decline in milk synthesis [46]. Moreover, maintenance requirements of energy also increased by 30% in HS dairy animal [47]. Therefore, energy intake would not be enough to cover the daily requirements for milk production. West [43] reported a reduction in DMI by 0.85 kg with every 1°C rise in air temperature above a cow’s TNZ, this decrease in intake accounts approximately 36% of the decrease in milk production [31]. According to Bouraoui *et al*. [42] daily THI negatively correlated to milk yield, as increase of THI value from 68 to 78 decreases DMI by 9.6% and milk production by 21%. Spiers *et al*. [44] reported that milk yield decreases by 0.41 kg/cow/day for each THI unit increase of above 69, feed intake decreased within a day after initiation of HS, while milk yield decreased after 2 days of HS. Gaafar *et al*. [48] reported that with increases in THI from 59.82 in the winter season to 78.53 in the summer season, HS reduced total (305 days) and daily milk yield by 39.00%, 31.40% and 29.84%, respectively. Total average milk production/cow was significantly (p<0.05) higher in spring period (42.74±4.98 L) compared to summer (39.60±5.091 L) [49]. Drop in milk production up to 50% in dairy animals might be due to reduced feed intake [9], whereas, rest could be reasons of metabolic adaptations to HS as HS response markedly alters post-absorptive carbohydrate, lipid, and protein metabolism a part of reduced feed intake [9]. Increased in basal insulin levels with improved insulin response in heat-stressed cows [9,12,46] and in ewes [50] were observed that explains the shift in glucose utilization in non-mammary gland tissue affecting milk synthesis [12]. HS during the dry period (i.e., last 2 months of gestation) reduced mammary cell proliferation and so, decreases milk yield in the following lactation [51]. Moreover, HS during the dry period negatively affects the function of the immune cell in dairy cows facing calving and also extended to the following lactation [51]. Singh *et al*. [52] also reported negative impacts of HS on lactation length, dry period, calving interval, milk constituents and milk yield in Murrah buffaloes. Upadhyay *et al*. [45] reported that the annual total milk loss due to thermal stress at the all-India level was 1.8 million tonnes or approximately 2% of the total milk production of the country amounting to a whopping Rs. 2661.62 crores per year. The negative impact of global warming on total milk production in India is also estimated to about 3.2 million tons by 2020 and more than 15 million tons by 2050.

The stage of lactation is an important factor for severity of imposed HS and animals which were in mid-lactation is mostly heat sensitive compared to early and late lactating counterparts [35,53]. The decline in milk production due to HS was 14% in early lactation and 35% in mid-lactation [35,53]. Average milk production in Holstein-Friesian during early lactation period (first 60 days of lactation) was significantly (p<0.05) higher in spring (42.74±4.98 L) than in summer (39.60±5.09 L) seasons [49]. Similar results were also observed [13] in early lactating dairy goats under HS with greater milk yield losses (9%) compared to late lactating animals (3%) and also reported greater reductions in milk fat at early lactation (12%) compared to late lactation (1%). Kumar *et al*. [10] also observed that high temperature with high humidity during calving had a detrimental effect on lactation yield and lactation length of Holdeo crossbred (Holstein Friesian × Deoni) cattle.

Hot and humid environment not only affects milk yield but also effects milk quality. Kadzere *et al*. [15] reported that milk fat, solids-not-fat (SNF) and milk protein percentage decreased by 39.7, 18.9 and 16.9%, respectively. Bouraoui *et al*. [42] observed lower milk fat and milk protein in the summer season. When THI value goes beyond 72, milk fat and protein content declines. In addition, the analysis of protein fractions also showed a reduction in percentages of casein, lactalbumin, immunoglobulin G (IgG) and IgA. 80% of these were associated with loss of productivity and 20% with health issues which might be due to disruption of internal homeostasis mechanism [54]. Zheng *et al*. [55] observed that HS significantly reduces the production of milk, the percentage of milk fat and percentage of proteins, but that it has no effect on the content of lactose in milk. Milk protein contents decline, whereas the response of milk fat content seems delayed and results are contradictory when THI is above 72 [53]. The content of proteins and fats was significantly (p<0.01) higher in spring period than in summer. However, values for percentage of lactose varied slightly (4.45±0.54% in spring vs. 4.03±0.24% in summer period; p>0.05) in these two seasons [49]. HS significantly reduced milk fat, protein, lactose, SNF and ash contents from 3.79%, 3.20%, 4.78%, 8.69%, 12.48% and 0.71% during the winter season to 3.49%, 3.07%, 4.59%, 8.34%, 11.83% and 0.67% during summer season [48]. A reduction in casein percentage and casein index (casein/total proteins ratio) was also decreased in summer (2.18% vs. 2.58% and 72.4% vs. 77.7%, respectively) compared to spring season [35,53]. Hamzaoui *et al*. [13] also reported milk with lower protein (6-13%) and lactose (1-5%) contents in HS goats. Continual genetic selection for greater performance results to increased HS sensitivity and decreasing trend in lactation curve as well as poor milk quality in of dairy animals during summer seasons.

### Effects on reproductive performance

High air temperature and humidity affects cellular functions by direct alteration and impairment of various tissues or organs of the reproductive system in both the sexes of the animal.

### Effects on female reproductive performance

#### Estrous period and follicular growth

HS reduces the length and intensity of estrus besides increases incidence of anestrous and silent heat in farm animals [6,52,56]. It increases ACTH and cortisol secretion [52], and blocks estradiol-induced sexual behavior [57]. Roth *et al*. [58] reported that developed follicles suffer damage and become non-viable when the body temperature exceeds 40°C. When female goats exposed to 36.8°C and 70% relative humidity for 48 h follicular growth to ovulation suppresses, accompanied by decreased LH receptor level and follicular estradiol synthesis activity [59]. Reduced granulosa cells aromatase activity and viability also contributed to poor estradiol secretion [59,60]. Low estradiol secretion suppresses signs of estrus, gonadotropin surge, ovulation, transport of gametes and ultimately reduced fertilization [60]. A temperature rise of more than 2°C in unabated buffaloes may cause negative impacts due to low or desynchronized endocrine activities particularly pineal-hypothalamo-hypophyseal-gonadal axis altering respective hormone functions [45]. They also reported that low estradiol level on the day of estrus during summer period may be the likely factor for poor expression of heat in Indian buffaloes [45].

#### Fertility

Multifactorial mechanisms involved in reducing fertility of dairy animals depending on the magnitude of HS. HS reduces oocyte development by affecting its growth and maturation [52]. It increases circulating prolactin level in animal’s results to acyclicity and infertility [52,61]. Moreover, 80% of estrus may be unnoticeable during summer [62] which further reduces fertility. A period of high-temperature results to increase secretion of endometrial PGF-2α, thereby threatening pregnancy maintenance leads to infertility [63]. Plasma follicle-stimulating hormone (FSH) surge increases and inhibin concentrations decrease during HS leading to variation in follicular dynamics and depression of follicular dominance that could be associated with low fertility of cattle during the summer and autumn [58]. However, FSH secretion is elevated under HS condition probably due to reduced inhibition of negative feedback from smaller follicles which ultimately affect the reproductive efficiency of dairy animals [64]. Conception rates were drop from about 40% to 60% in cooler months to 10-20% or lower in summer, depending on the severity of the thermal stress [65]. About 20-27% drop in conception rates [66] or decrease in 90-day non-return rate to the first service in lactating dairy cows were recorded in summer [67]. Amundson *et al*. [68] reported a reduction (p<0.01) in pregnancy rate in summer (62%) and decreasing in spring (44%) when the average daily minimum temperature and average daily THI were equal to or above 16.7°C and 72.9 respectively. Moreover, severe HS, only 10-20% of inseminations were resulted, in normal pregnancies, were also reported [58]. Oocytes of cows exposed to thermal stress lose their competence for fertilization [69] and development to the blastocyst stage [70]. Recently, Lacerda and Loureiro [71] also reported HS decreases fertility by diminishing quality of oocytes and embryos through direct and indirect effects.

#### Embryonic growth and development

Embryonic growth and survival also affected during thermal stress in dairy animals. HS causes embryonic death by interfering with protein synthesis [72], oxidative cell damage [60], reducing interferon-tau production for signaling pregnancy recognition [63] and expression of stress-related genes associated with apoptosis [73]. Low progesterone secretion limits endometrial function and embryo development [60,64]. Exposure of lactating cows to HS on the 1^st^ day after estrus reduced the proportion of embryos that developed to the blastocyst stage on the day 8^th^ after estrus [74]. Further, exposure of post-implantation embryos (early organogenesis) and fetus to HS also leads to various teratologies [60]. The deleterious effects of HS in the embryo are most evident in early stages of its development [75]. However, embryos subjected to high temperatures *in vitro* or *in vivo* until day 7 of development (blastocyst) showed lower pregnancy rates at day 30 [75] and higher rates of embryonic loss on day 42 of gestation [75] and lactation yield as well as postpartum ovarian activity. Fetal malnutrition and eventually fetal growth retardation under thermal stress were also reported [6,51].

### Effects on male reproductive performance

Bull is recognizing as more than half of the herd and hence, bull’s fertility is equally or more important for fertilization of oocyte to produce a good, viable and genetically potential conceptus. It is well known that bull testes must be 2-6°C cooler than core body temperature for fertile sperm to be produced. Therefore, increased testicular temperature results from thermal stress could changes in seminal and biochemical parameters leads to infertility problems in bulls. The significant seasonal difference in semen characteristics was reported by several studies [76-80]. Cardozo *et al*. [76] reported seasonal effects on changes in testicular volume, hormonal profiles, sexual behavior and semen quality that affect the reproductive performance of males. Balic *et al*. [77] studied seasonal influence on 19 *Bos taurus* (simmental) bulls and found summer HS declined semen quality parameters. They also reported that younger bulls are more sensitive to elevated air temperatures during the summer seasons. Mishra *et al*. [78] in a study observed that membrane integrity status of fresh spermatozoa in four different breeds of bulls (crossbred, Red Sindhi, Haryana and Jersey) were affected significantly (p<0.01) with increases in air temperature from 10 to 18°C to more than 35°C. Rahman *et al*. [79] also reported that HS spermatozoa showed a highly reduced (p<0.01) fertilization rate in comparison to non-HS or normal control spermatozoa (53.7% vs. 70.2% or 81.5%, respectively). Bhakat *et al*. [80] observed optimal semen qualities during winter, poor during summer and intermediate during rainy season and conclude that hot-dry or summer season adversely affect the various bio-physical characteristics of semen in Karan Fries bulls. Hence, HS significantly lowers conception as well as fertility rates per insemination of male and subsequently reduces male’s fitness.

## Strategies for Ameliorating HS

Physical modifications of the environment, nutritional management and genetic development of thermotolerance breeds are key components for sustainable livestock production in tropical hot climates [81].

### Physical modifications of environment

The most common approach to ameliorate HS is to alter the cow’s environment through provision of house or shade (along with feed and drinking water), evaporative cooling system with water in the form of fog, mist or sprinkling with natural or forced air movement, and possibly cooling ponds [81]. Modification of microenvironment to enhance heat dissipation mechanism to relieve HS is one of the most important measures to be considered in hot environment. Cooling ponds and sprinklers can also be used to cool the environment. Cooling can also improve reproductive performance in cows and heifers, and probably, the most effective cooling systems currently in use are those that couple evaporative cooling with tunnel ventilation or cross ventilation [6]. Dairy cattle allowed access to sprinklers (with and without forced ventilation) have increased milk production, improved reproduction and improved conversion of feed to milk [82]. Shading is one of the cheapest ways to modify an animal’s environment during hot weather. For outdoors animals, provision of shade (natural or artificial) is one of the simplest and cost-effective methods to minimize heat from solar radiations. Trees are very effective and natural shading materials providing shade to the animals combined with beneficial cooling as moisture evaporates from the leaves. Artificial shades can be used to protect from the effects of solar radiation in absence of natural shade. Various types of roofing materials can be used from metal to synthetic materials for shade structures among which a white galvanized or aluminum roof is considered best.

### Nutritional management

Nutritional modifications could help animals to maintain homeostasis or prevent nutrient deficiencies that result from HS. Lower DMI during hot weather reduces nutrients available for absorption, and absorbed nutrients are used less efficiently [83]. Rations should be >18% protein on a dry basis as overfeeding requires more energy to excrete excess nitrogen as urea. Optimizing ruminally undegraded protein improves milk yield in hot climates [83]. DMI and milk yield increased for cows fed with diets containing 14% versus 17 or 21% acid detergent fiber (ADF). However, milk yield was less sensitive to change in dairy temperature for cows fed with 14% ADF diet [43]. Increasing dietary fat content enhanced milk production efficiency and yield in the warm season [84]. Feed containing low fiber rations during hot weather is logical since heat production is highly associated with metabolism of acetate compared with propionate [81]. HS causes oxidative damage which could be minimized through supplementation of vitamins C, E and A and also mineral such as zinc [85]. Vitamin E acts as an inhibitor – “chain blocker”- of lipid peroxidation and ascorbic acid prevents lipid peroxidation due to peroxyl radicals. It also recycles vitamin E, vitamin C and zinc are known to scavenge ROS during oxidative stress. Further, vitamin C assist in the absorption of folic acid by reducing it to tetrahydrofolate, the latter again acts as an antioxidant. Use of vitamin C along with electrolyte supplementation was found to relieve the animals of oxidative stress and boosts cell-mediated immunity in buffaloes [86]. West [83] reported that Na^+^ and K^+^ status of the body stayed normal during HS when supplemented with electrolytes could be due to better regulation of acid-base balance in the blood. Yeast product supplementation plays an important role in digestibility of nutrient by altering the volatile fatty acids production in the rumen, decrease the production of ruminal ammonia and increase in ruminal microorganism population. Live yeast was also reported as beneficial to small ruminant nutrition and production [87].

### Genetic selection

Advances in environmental modifications and nutritional management in part alleviate the impact of thermal stress on animal performance during the hotter seasons. However, long-term strategies have to be evolved for adaptation to climate change. Differences in thermal tolerance exist between livestock species provide clues or tools to select thermotolerant animals using genetic tools. The identification of heat-tolerant animals within high-producing breeds will be useful only if these animals are able to maintain high productivity and survivability when exposed to HS conditions. Cattle with shorter hair, hair of greater diameter and lighter coat color are more adapted to hot environments than those with longer hair coats and darker colors [53]. This phenotype has been characterized in *B. taurus* tropical cattle (senepol and carona), and this dominant gene is associated with an increased sweating rate, lower RT and lower RR in homozygous cattle under hot conditions [88]. There is heat shock gene related to thermotolerance that was identified and being used as marker in marker assisted selection and genome-wide selection to developed thermotolerant bull that are used in breeding program. Major families of Hsps are Hsp100, Hsp90, Hsp70, Hsp60, Hsp40 and the small Hsps (so-called Hsps of sizes below 30 kDa). HSPs have a critical role in the recovery of cells from stress and in cytoprotection as well as guarding cells from subsequent insults. Hsp gene expression under thermal stress changes include: (i) Activation of heat shock transcription factor 1 (HSF1); (ii) increased expression of Hsp genes and decreased expression and synthesis of other proteins; (iii) increased glucose and amino acid oxidation and reduced fatty acid metabolism; (iv) endocrine system activation of the stress response; and (v) immune system activation via extracellular secretion of Hsp. If the stress persists, these gene expression changes lead to an altered physiological state referred to as “acclimation,” a process largely controlled by the endocrine system [8]. Several reports showed associations of SNP in the Hsp genes with thermal stress response and tolerance in farm animals. Association of polymorphisms in Hsp90AB1 with heat tolerance has also been reported in Thai native cattle [89], Sahiwal and Frieswal cattle [90], HSF1 gene [91], HSP70A1A gene [92], HSBP1 [93] in Chinese Holstein cattle. There are non-Hsps genes also revealed to undergo changes in expression in response to HS. For example ATP1B2 gene in Chinese Holstein cows [94] and ATP1A1 gene in jersey crossbred cows [95] was observed to have associated with thermotolerance. These SNPs could be used as markers in marker assisted selection to developed thermotolerant animal in early ages. Further, thermotolerant bull can be used in breeding policy to have thermal adapted offspring.

## Conclusion

Extended periods of high air temperature coupled with high relative humidity compromise the ability of dairy animal to dissipate excess body heat which affects feed intake, milk production, and reproductive efficiency and ultimately reducing profitability for dairy farmers. However, by minimizing body temperature, greater feed intake could be encouraged. Moreover, the gross efficiency with which dietary nutrients are used by the cow for performance could also be improved. The loss of electrolytes via skin secretions has to be minimized by improvement of housing and cooling of the animals. Standardization of mineral supplement to control acid-base balance should be considered in animal under different level of thermal stress. Increase pregnancy rate of HS cows could be achieved by improving various managemental conditions. Identification of genes associated with thermotolerance and using these genes as markers in the breeding program or marker assisted selection should be applied to identify animals adapted to thermal stress considering genotype-environment interactions (G × E) in addition to higher productivity. Further research on climate resilient animal agriculture is the need of the hour for sustainability in dairy farming system, especially in hot humid climatic regions.

## Authors’ Contributions

RD prepared the initial version of the manuscript. LS, NV, PB, JS and RK assisted in literature collection. RD and IM drafted and revised the manuscript for critical scientific corrections. All authors read and approved the final manuscript.
